# Relationship between waist circumference and cardiorespiratory fitness in Chinese children and adolescents: Results from a cross-sectional survey

**DOI:** 10.1016/j.jesf.2021.10.004

**Published:** 2021-11-03

**Authors:** Yuan Liu, Xiaojian Yin, Feng Zhang, Yuqiang Li, Cunjian Bi, Yi Sun, Ming Li, Ting Zhang

**Affiliations:** a Key Laboratory of Adolescent Health Assessment and Exercise Intervention of the Ministry of Education, East China Normal University, Shanghai, 200241, China; b College of Physical Education and Health, East China Normal University, Shanghai, 200241, China; c College of Economics and Management, Shanghai Institute of Technology, Shanghai, 201418, China

**Keywords:** Waist circumference, Cardiorespiratory fitness, Children and adolescents, Maximal oxygen consumption, 20 m shuttle Run test

## Abstract

**Background:**

This article assessed the relationship between waist circumference (WC) and cardiorespiratory fitness (CRF) of children and adolescents aged 7–18 years.

**Methods:**

Using a stratified cluster random sampling method, 92,574 children and adolescents (47,364 males and 45,210 females) were extracted. CRF was measured by performance in the 20 m shuttle run test (20mSRT) and the subsequent estimation of maximal oxygen consumption ($\dot{\text{V}}$O_2max_) using the Léger equations. Participants were divided into five groups of WC percentiles and three groups of CRF percentiles by the Lambda Mu Sigma (LMS). The correlation between WC and CRF was examined by one-way ANOVA and curvilinear regression analysis.

**Results:**

WC increased with age, while $\dot{\text{V}}$O_2max_ showed an age-related decline. Controlling for gender, urban, and rural factors, for children and adolescents aged 10–12, 13–15, and 16–18 years, the $\dot{\text{V}}$O_2max_ Z-score of the normal WC group was significantly higher than the very low WC group (*P* < 0.05). Controlling for gender, urban, and rural factors, for participants aged 7–18 years, the $\dot{\text{V}}$O_2max_ Z-score of the normal WC group was significantly higher than the high WC group and the very high WC group (*P* < 0.05).

**Conclusions:**

It generally shows a “parabolic” trend between WC-Z and $\dot{\text{V}}$O_2max_-Z. The CRF among children and adolescents in the normal WC group is significantly higher than that in the low and the high WC groups.

## 1 Introduction

Cardiorespiratory fitness (CRF) is a core element of all components of physical fitness in children and adolescents.^1^^,^^2^ CRF is the ability of the respiratory, skeletal muscle, and circulatory systems to supply oxygen for energy transfer to support muscle activity during physical activity. The population in China has experienced raised living standards and substantial lifestyle changes with the rapid development of the economy. Obesity rates have risen sharply, but declines in health have become a serious problem for society. The rise in obesity prevalence and declining physical health among children and adolescents have become a major social problem.^3^^,^^4^ Obesity, which parameter is BMI, WC et al. plays a central role in the association between CRF and cardiometabolic risk (CMR) factors.^5^^,^^6^ Furthermore, the association between obesity and CMR is also influenced by CRF.^6^ A study showed that approximately 100 million children and adolescents around the world were obese in 2015, with the highest number (approximately 15.3 million) in China.^7^ According to a report on childhood obesity in China, from 1985 to 2014, the prevalence of overweight among children and adolescents increased from 2.1% to 12.2%, with the prevalence of obesity increasing from 0.5% to 7.3%.^8^ These prevalence estimates for overweight and obesity are mainly based on body mass index (BMI) diagnostic indicators, but, BMI can not differentiate between the distribution of muscle and abdominal fat.^9^

Waist circumference (WC) is an effective indicator of abdominal obesity^10^^,^^11^ that reflects the accumulation of fat in the abdomen and has shown to be valid predictor of future cardio-metabolic and chronic diseases.^12^^,^^13^ A study of the association between change in WC status over 2 years on left ventricular hypertrophy (LVH) found that children who maintained normal waist circumference had reduced odds for developing LVH.^14^ Furthermore, WC had a slightly higher correlation with cardiovascular risk factors than BMI,^15^ and the strongest correlation with insulin and systolic blood pressure in Chinese children.^16^ Many people have “insidious” obesity that is characterized by a BMI in the normal range but particularly high abdominal fat accumulation.^17^ Abdominal adiposity is positively associated with risk for metabolic disease and independent of total body adiposity.^18^ In recent years, the prevalence of high WC in children and adolescents has been increasing worldwide.^19^, ^20^, ^21^ Meanwhile, CRF levels decreased significantly in children and adolescents in China.^22^ A study reported that the CRF trend of children and adolescents aged 9–17 years found a substantial international decline between 1981 and 2000, however, the international trend in CRF has been diminishing and stabilizing with a negligible change between 2000 and 2014 from upper-middle and high-income countries.^23^ Chinese scholars demonstrated that CRF of children and adolescents was generally worse in China than in Japan.^24^ In conclusion, the number of children with high waist circumference is increasing, while CRF level is decreasing. Based on the current situation, it is particularly important to explore the relationship between waist circumference and CRF. Most studies focused on the relationship between BMI and CRF,^25^^,^^26^ but there were a few studies on WC and CRF.

To summarize, the correlation between body composition and CRF in children and adolescents has been examined, but most studies have focused on the relationship between BMI and CRF. However, the research on the relationship between WC and CRF was minimal, and previous studies have used local and small samples that were not representative of Chinese children and adolescents. In this study, 92,574 children and adolescents were examined to analyze the relationship between WC and CRF. The significance of this article was to provide a theoretical basis and empirical support for the study of the body physical health of children and adolescents in China.

## 2 Materials and methods

### 2.1 Participants and sampling

A stratified random cluster sampling method was used to select participants from a cross-sectional survey called “Formulation of new methods and evaluation criteria for the physical health of children and adolescents in China” in 2015–2016. Considering the population weight and geographical location, we used the proportion of each index to sample the main data bulletin of the sixth national census in 2010.^27^ There are six main traditional administrative regions in China (east, north, central south, northwest, southwest, and northeast). After excluding invalid data and extreme values, based on the population ratio of about 1:1 in males and females, 92,574 participants (47,364 males and 45,210 females) were collected for the current study (Table 1). The participants who were enrolled in full-time school, had no physical disability, no major psychological condition, and were able to participate in the CRF test, were included in this study. The survey was approved by the Human Experiment Ethics Committee of East China Normal University (approval No. HR2016/12055). All participants were informed about survey requirements before data collection. The participants' names have been numerically coded to avoid revealing personal information. Researchers carefully checked the health of the participants to determine whether they met the health standards of the test.

**Table 1:** Sample distribution of children and adolescents aged 7–18 years in China.

Age (year)	Males N(%)	Females N(%)	Total
**7**	4996(53.3)	4371(47.7)	9367
**8**	3460(52.0)	3190(48.0)	6650
**9**	3828(50.9)	3691(49.1)	7519
**10**	4093(53.0)	3629(47.0)	7722
**11**	4106(53.3)	3604(46.7)	7710
**12**	3837(52.2)	3518(47.8)	7355
**13**	3783(52.7)	3396(47.3)	7179
**14**	3803(51.7)	3552(48.3)	7355
**15**	4164(51.0)	4009(49.0)	8173
**16**	4229(49.4)	4337(50.6)	8566
**17**	3599(47.8)	3936(52.2)	7535
**18**	3466(46.6)	3977(53.4)	7443
**Total**	47364(51.2)	45210(48.8)	92574

### 2.2 Waist circumference

WC was tested according to the implementation rules in the 2014 national survey report on students physical health.^28^ Participants stood upright, with their arms crossed in front of the chest and their feet together, so that their weight was evenly distributed between their feet and their abdominal skin was exposed. During the test, subjects were asked to breathe gently. The testers faced the participants and put the nylon tape (*Hoblemanss*) 1 cm (cm) above the navel and placed it in a horizontal plane around the waist. The line of sight was on the same level as the nylon tape, and the reading was in cm (accuracy: one decimal place). The participants were measured twice and averaged to ensure the accuracy of the experiment. The test error could not exceed 0.1 cm.

### 2.3 Cardiorespiratory fitness

The FitnessGram protocol was used to conduct the 20 m shuttle run test (20mSRT).^29^ The test was conducted on rubberized school playgrounds or covered stadiums, in which two lines 20-m apart were drawn. The required equipment consisted of an audio player and the beep test audio recordings. After participants had adequately warmed up and viewed an instructional 20mSRT video recorded in advance, participants were asked to continually run between the two lines 20 m apart, turning when signaled by the recorded beeps. The method is as follows: Participants started at a speed of 8.0 km h^−1^. After approximately 1 min, at the end of the first stage on the cassette called "stage 1", a sound indicated an increase in speed to 9.0 km h^−1^. Thereafter, the speed was increased by 0.5 km h^−1^ each minute. Children ran in time with a series of audible signals for as long as possible until they could no longer run the 20 m distance in time with the audio signal (on two consecutive occasions) or when they stopped because of volitional fatigue. The last lap completed (not necessarily the level stopped at) was recorded as the result. Factors that may affect the reliability of the test results (e.g., test motivation, test climate and test environment conditions) were strictly controlled.

In this study, $\dot{\text{V}}$O_2max_ (mL·kg^−1^·min^−1^) was calculated using the equation developed by Léger.^30^ The last birthday of each participant was taken as the standard for calculating the age, and the speed of the last completion stage (km·h^−1^) was calculated.

$$\dot{\text{V}}{{\text{O}}_{2}}_{\text{max}}\left({\text{mL·kg}}^{-1}{\text{·min}}^{-1}\right)\;=\text{ 31.025 }+\;\left(3.238\text{ × S}\right)\;-\;\left(3.248\text{ × A}\right)\;+\;\left(0.1536\text{ × S × A}\right)$$

S = the running speed at the last completed stage in km·h^−1^; S = 8 + (0.5 × completed stage number).
A = age at the last birthday.

### 2.4 Statistical analysis

The participants were categorized according to age and gender. WC was categorized according to percentile by the Lambda Mu Sigma (LMS): very low (WC < *P*_5_), low (*P*_5_ ≤ WC < *P*_15_), normal (*P*_15_ ≤ WC < *P*_85_), high (*P*_85_ ≤ WC < *P*_95_), and very high (WC ≥ *P*_95_). According to percentile, CRF was divided into low (CRF < *P*_25_), normal (*P*_25_ ≤ CRF ≤ *P*_85_) and high (CRF > *P*_85_). In addition, children and adolescents were categorized into four age groups^25^: lower primary school age (7–9 years); upper primary school age (10–12 years), middle school age (13–15 years), and high school age (16–18 years). The mean and standard deviation (SD) of WC and $\dot{\text{V}}$O_2max_ were calculated according to age and gender. WC Z-score and $\dot{\text{V}}$O_2max_ Z-score were calculated by gender and age, respectively. Z-score = (measured value - mean)/standard deviation.

One-way analysis of variance (ANOVA) was used to compare the $\dot{\text{V}}$O_2max_ Z-score of children and adolescents with different WC groups. A general linear model was used to examine the difference in $\dot{\text{V}}$O_2max_ Z-score between age groups in children and adolescents after adjusting urban-rural differences. A curvilinear regression analysis model was established with WC Z-score as independent variables and $\dot{\text{V}}$O_2max_ Z-score as dependent variables. The relationships between WC Z-score and $\dot{\text{V}}$O_2max_ Z-score for different age groups (7–9 years, 10–12 years, 13–15 years, and 16–18 years) were investigated using a nonlinear regression model ($\dot{\text{V}}$O_2max_-Z = a (WC-Z)^2^+ b (WC-Z)+ c; where a, b, and c were constants). The effect of the difference between the low group and the high group was calculated using Cohen's d (small effect = 0.2; medium effect = 0.5; large effect = 0.8).^31^ Data were analyzed using IBM SPSS 25.0 software (IBM Corp., Armonk, NY, USA), the test level was α = 0.05. It was considered significant if *P* < 0.05.

## 3 Results

### 3.1 Descriptive characteristics for waist circumference and $\dot{V}$O_2max_

Table 2 showed the mean and standard deviation of WC and $\dot{\text{V}}$O_2max_ of children and adolescents in different ages. WC of both males and females increased with age. However, $\dot{\text{V}}$O_2max_ showed an age-related decline. The score of the WC and $\dot{\text{V}}$O_2max_ was higher in males than in females. The $\dot{\text{V}}$O_2max_ of 7–9 years was the highest (47.3 ml kg^−1^·min^−1^). Aged 10–12-year-olds and 16–18-year-olds, the proportion of the moderate CRF group ($\dot{\text{V}}$O_2max_ ≥ percentile 25 and < percentile 85) was highest (Table 2).

**Table 2:** Descriptive data of waist circumference and $\dot{\text{V}}$O_2max_ in children and adolescents.

Age group	Gender	Age^a^	WC^b^	Estimated $\dot{\text{V}}$O_2max_^c^	CRF Group (%)		
					Low^d^	Moderate^e^	High^f^
**7**–**9**	**Males**	7.9 ± 0.84	57.2 ± 11.3	47.4 ± 2.6	35.5	36.1	28.4
	**Females**	7.9 ± 0.84	55.3 ± 9.5	47.1 ± 2.4	35.9	32.7	31.4
	**Total**	7.9 ± 0.84	56.3 ± 10.5	47.3 ± 2.5	35.7	34.5	29.8
**10**–**12**	**Males**	11.0 ± 0.81	65.9 ± 11.1	44.6 ± 3.6	16.0	57.8	26.2
	**Females**	11.0 ± 0.82	62.9 ± 9.3	43.9 ± 3.1	16.9	48.3	34.8
	**Total**	11.0 ± 0.81	64.5 ± 10.4	44.3 ± 3.4	16.4	53.4	30.2
**13**–**15**	**Males**	14.0 ± 0.82	71.1 ± 10.8	43.8 ± 5.2	32.3	18.3	49.4
	**Females**	14.1 ± 0.82	66.3 ± 8.4	40.7 ± 4.2	25.0	39.8	35.1
	**Total**	14.0 ± 0.82	68.8 ± 10.0	42.3 ± 5.0	28.8	28.7	42.5
**16**–**18**	**Males**	16.9 ± 0.83	74.3 ± 10.1	40.6 ± 6.1	24.2	39.6	36.2
	**Females**	17.0 ± 0.82	67.3 ± 7.6	35.5 ± 4.4	13.2	62.1	24.7
	**Total**	17.0 ± 0.82	70.6 ± 9.5	37.9 ± 5.9	18.5	51.3	30.2

Note: ^a^, in years; ^b^, in cm; ^c^, in ml/kg/min; ^d^, $\dot{\text{V}}$O_2max_ < percentile 25; ^e^, $\dot{\text{V}}$O_2max_ ≥ percentile 25 and < percentile 85; ^f^, $\dot{\text{V}}$O_2max_ ≥ percentile 85.

WC, waist circumference; CRF, cardiorespiratory fitness.

**Fig. 1:**
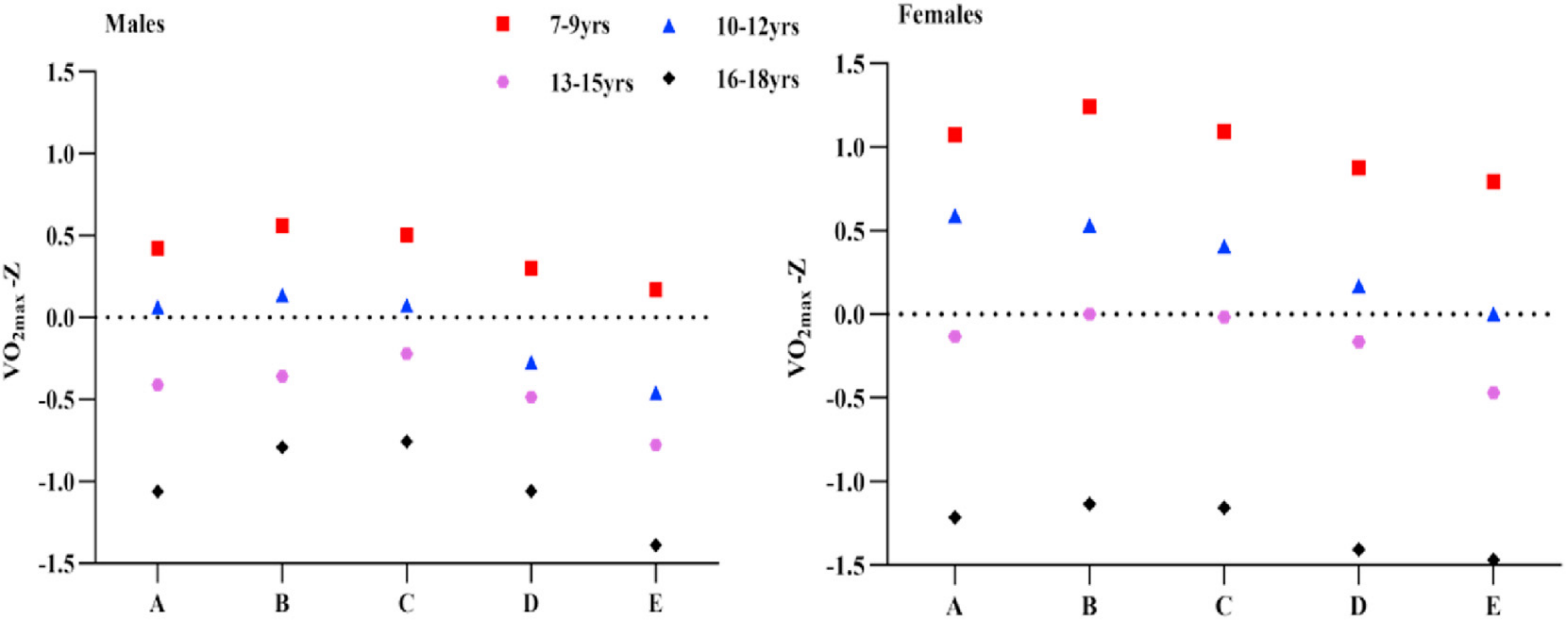
$\dot{\text{V}}$O_2max_ of Chinese children and adolescents aged 7–18 years in different waist circumference groups. Note: A = waist circumference < percentile 5; B = waist circumference ≥ percentile 5 and < percentile 15; C = waist circumference ≥ percentile 15 and < percentile 85; D = waist circumference ≥ percentile 85 and < percentile 95; E = waist circumference ≥ percentile 95.

Fig. 1 showed that the $\dot{\text{V}}$O_2max_ Z-score of children and adolescents in all age groups first increased and then decreased with an increase of WC percentile. The results for the very low WC group and the low WC group showed that the $\dot{\text{V}}$O_2max_ of males and females aged 7–9 years and 10–12 years changed little with WC, whereas the $\dot{\text{V}}$O_2max_ of males and females aged 13–15 years and 16–18 years changed more significantly.

Age, WC, and Estimated $\dot{\text{V}}$O_2max_ values are given as the mean ± SD. CRF Group values are given as percentage.

### 3.2 Comparison of $\dot{V}$O_2max_ Z-score in different waist circumference groups

In males aged 7–9, 10–12 and 13–15 years, the $\dot{\text{V}}$O_2max_ Z-score of the normal WC group was significantly higher than that of the low WC group (*P* < 0.05). In males aged 7–18 years, the $\dot{\text{V}}$O_2max_ Z-score of the normal WC group was significantly higher than that of the high WC group (*P* < 0.05). In males aged 10–12, 13–15 and 16–18 years, the average $\dot{\text{V}}$O_2max_ Z-score of those with normal WC was higher than the other four WC groups. In females aged 7–9, 13–15 and 16–18 years, the $\dot{\text{V}}$O_2max_ Z-score of those with low WC was higher than the other WC groups. In females aged 10–12 and 13–15 years, the $\dot{\text{V}}$O_2max_ Z-score of those with normal WC was significantly higher than those with very low WC group (*P* < 0.05). Controlling for gender differences, for children and adolescents aged 10–12, 13–15, and 16–18 years, the $\dot{\text{V}}$O_2max_ Z-score of the normal WC group was significantly higher than the very low WC group (*P* < 0.05). Controlling for gender differences, for participants aged 7–18 years, the $\dot{\text{V}}$O_2max_ Z-score of the normal WC group was significantly higher than the high WC group and the very high WC group (*P* < 0.05). Controlling for gender differences, the highest $\dot{\text{V}}$O_2max_ Z-scores were observed aged 7–9 years with the low WC group (Table 3).

**Table 3:** Comparison of $\dot{\text{V}}$O_2max_ Z-score in different waist circumference groups in children and adolescents aged 7–18 years in China.

Project	Age Group	Percentile Group A		Percentile Group B		Percentile Group C		Percentile Group D		Percentile Group E		Cohen's d #									
		N	Mean (SD)	N	Mean (SD)	N	Mean (SD)	N	Mean(SD)	N	Mean (SD)	A/B	A/C	A/D	A/E	B/C	B/D	B/E	C/D	C/E	D/E
**Males**	**7-9yrs**	618	0.42(0.56)	1255	0.56(0.65)	8707	0.51(0.68)	1168	0.30(0.65)	536	0.17(0.68)	0.2∗	0.1∗	0.2∗	0.4∗	0.1∗	0.4∗	0.6∗	0.3∗	0.5∗	0.2∗
	**10**–**12yrs**	673	0.07(0.78)	1146	0.14(0.75)	8569	0.08(0.79)	1080	−0.27(0.71)	568	−0.46(0.67)	0.1∗	0.0	0.5∗	0.7∗	0.1∗	0.6∗	0.8∗	0.5∗	0.7∗	0.3∗
	**13**–**15yrs**	631	−0.41(0.92)	1258	−0.36(0.85)	8096	−0.22(0.90)	1223	−0.49(0.84)	542	−0.78(0.81)	0.1	0.2∗	0.1	0.4∗	0.2∗	0.2∗	0.5∗	0.3∗	0.6∗	0.4∗
	**16**–**18yrs**	805	−1.06(1.06)	1061	−0.79(1.1)	7814	−0.76(1.1)	1058	−1.06(1.0)	556	−1.39(0.91)	0.3∗	0.3∗	0.0	0.3∗	0.0	0.3∗	0.6∗	0.3∗	0.6∗	0.3∗
**Females**	**7-9yrs**	582	1.07(0.74)	1198	1.24(0.89)	7912	1.1(0.82)	1017	0.88(0.80)	543	0.79(0.92)	0.2∗	0.0	0.3∗	0.3∗	0.2∗	0.4∗	0.5∗	0.3∗	0.3∗	0.1
	**10**–**12yrs**	762	0.59(0.88)	845	0.53(0.77)	7635	0.41(0.81)	997	0.17(0.79)	512	0.003(0.73)	0.1	0.2∗	0.5∗	0.7∗	0.2∗	0.5∗	0.7∗	0.3∗	0.5∗	0.2∗
	**13**–**15yrs**	581	−0.13(0.95)	1099	0.01(1.0)	7940	−0.02(1.0)	805	−0.16(1.0)	532	−0.47(0.93)	0.1∗	0.1∗	0.0	0.4∗	0.0	0.2∗	0.5∗	0.1∗	0.5∗	0.3∗
	**16**–**18yrs**	746	−1.21(1.2)	1545	−1.13(1.2)	8353	−1.16(1.3)	717	−1.41(1.3)	889	−1.47(1.2)	0.1	0.0	0.2∗	0.2∗	0.0	0.2∗	0.3∗	0.2∗	0.3∗	0.1
**Total**	**7-9yrs**	1200	0.74(0.73)	2453	0.89(0.85)	16619	0.79(0.80)	2185	0.57(0.78)	1079	0.48(0.87)	0.2∗	0.1	0.2∗	0.3∗	0.1∗	0.4∗	0.5∗	0.3∗	0.4∗	0.1∗
	**10**–**12yrs**	1435	0.34(0.88)	1991	0.31(0.79)	16204	0.23(0.80)	2077	−0.06(0.79)	1080	−0.24(0.74)	0.0	0.1∗	0.5∗	0.7∗	0.1∗	0.5∗	0.7∗	0.4∗	0.6∗	0.2∗
	**13**–**15yrs**	1212	−0.28(0.95)	2357	−0.19(0.94)	16036	−0.12(0.96)	2028	−0.36(0.92)	1074	−0.62(0.88)	0.1∗	0.2∗	0.1∗	0.4∗	0.1∗	0.2∗	0.5∗	0.3∗	0.5∗	0.3∗
	**16**–**18yrs**	1551	−1.13(1.13)	2606	−0.99(1.2)	16167	−0.96(1.2)	1775	−1.20(1.1)	1445	−1.44(1.1)	0.1∗	0.1∗	0.1	0.3∗	0.0	0.2∗	0.4∗	0.2∗	0.4∗	0.2∗

Note: ^#^effect size between different groups ∗ *P* < 0.05. A = waist circumference < percentile 5; B = waist circumference ≥ percentile 5 and < percentile 15; C = waist circumference ≥ percentile 15 and < percentile 85; D = waist circumference ≥ percentile 85 and < percentile 95; E = waist circumference ≥ percentile 95.

**Fig. 2:**
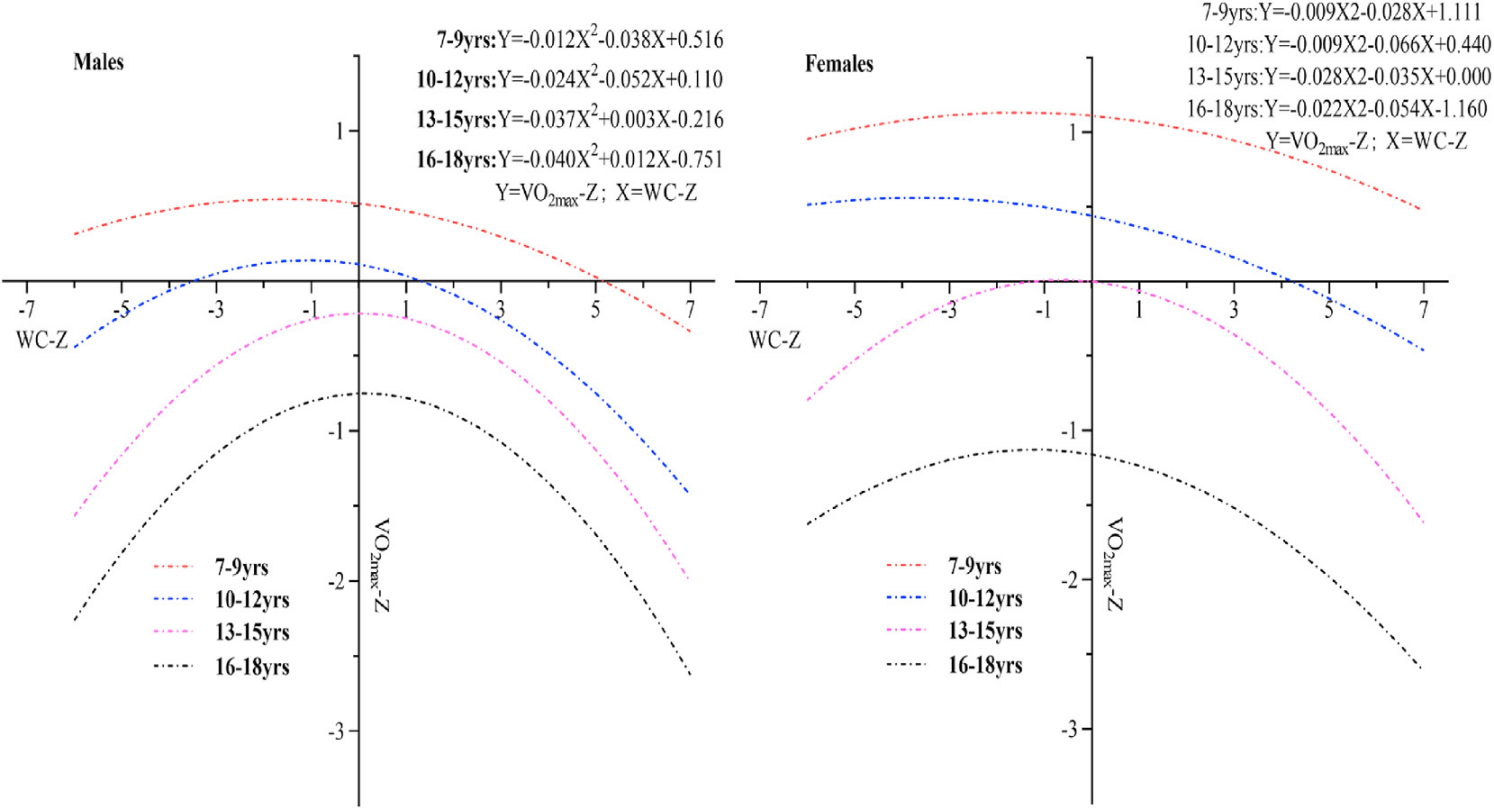
Relationship between WC-Z-score and $\dot{\text{V}}$O2max Z-score of children and adolescents.

Fig. 2 showed the relationship between WC Z-score and $\dot{\text{V}}$O_2max_ Z-score in children and adolescents of different genders and ages. Among them, the $\dot{\text{V}}$O_2max_ Z-score of females showed a slight increase first and then decreased, while the $\dot{\text{V}}$O_2max_ Z-score for males increased first and then decreased rapidly as the WC Z-score increased. The parabolic curve of females was more gentle than that of males. Both males' and females' $\dot{\text{V}}$O_2max_ Z-score showed a trend that is a slight increase first and then decreased with the WC Z-score increased. The relationship between WC Z-score and $\dot{\text{V}}$O_2max_ Z-score showed a “parabolic” trend (an inverted U shape).

### 3.3 Comparison on 20mSRT (laps) performance between Chinese and the international standards

Table 4 showed the comparison on 20mSRT (laps) performance between Chinese children and adolescents and international standards. The number of lap on 20mSRT increased with age. In males, Chinese children and adolescents aged 9–17 years performed worse than their international peers, with differences ranging from 4 to 9 laps. Except for the 9–11 years age groups, Chinese females outperformed their international peers, with the largest positive performance difference of 4 laps at 13 and 14 years old. (Table 4).

**Table 4:** Comparison on 20mSRT (laps) performance between Chinese and the international standards.

Gender	Age(yr)	China		International^a^		Difference
		N	Mean	N	Mean	
Males	9	3828	23	101532	32	−9
	10	4093	25	81538	33	−8
	11	4106	28	79660	36	−8
	12	3837	33	70266	39	−6
	13	3783	40	72913	44	−4
	14	3803	44	65317	48	−4
	15	4164	48	53287	52	−4
	16	4229	48	35490	54	−6
	17	3599	50	29123	57	−7
Females	9	3691	21	98166	26	−5
	10	3629	23	81415	27	−4
	11	3604	26	78029	28	−2
	12	3518	29	69497	28	1
	13	3396	33	92491	29	4
	14	3552	33	55035	29	4
	15	4009	33	43730	30	3
	16	4337	31	34978	30	1
	17	3936	32	29559	30	2

a Tomkinson GR, Lang JJ, Tremblay MS et al. International normative 20 m shuttle run values from 1 142 026 children and youth representing 50 countries. Br J Sports Med. 2017;51(21):1545-1554.

**Fig. 3:**
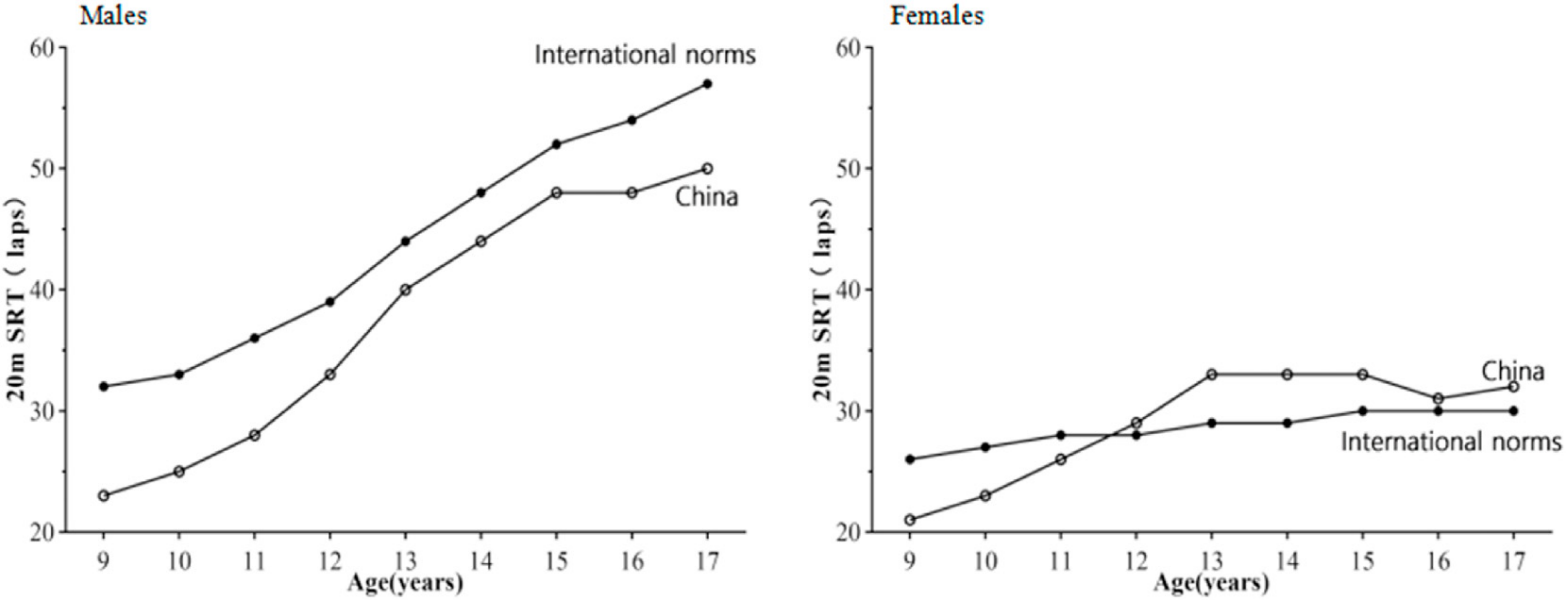
Standardized differences in 20mSRT (number of completed shuttles) performance between age-matched boys and girls for Chinese children and adolescents compared with the international values.

Fig. 3 showed the standardized differences in 20mSRT (laps) performance between age-matched males and females for Chinese children and adolescents compared with international values. Chinese males consistently outperformed females in 20mSRT (laps). In males, the mean 20mSRT performance of Chinese children and adolescents was worse than the international mean. Mean 20mSRT performance for Chinese females was worse than the international mean for 9- to 11-year-olds, but better for the 12- to 17-year-olds. (Fig. 3).

## 4 Discussion

In this study, we observed that the score of the WC and $\dot{\text{V}}$O_2max_ was higher among males than among females. This study found that both males' and females' $\dot{\text{V}}$O_2max_ showed a “parabolic” trend. The parabolic curve of $\dot{\text{V}}$O_2max_ was more gentle in females than in males. The results were in agreement with previous studies. Some similar studies have shown that the relationship between WC and CRF is related to gender.^32^^,^^33^ $\dot{\text{V}}$O_2max_ Z-score was the highest when the WC Z-score was within the normal range. In other words, the normal WC group showed better CRF levels than the low WC group or high WC group. Moreover, many studies have proved that 20mSRT is one of the most widely used in CRF tests.^34^^,^^35^ A previous study of the 20mSRT reported a test-retest reliability coefficient of 0.89 for children.^30^ Besides, the 20mSRT can authentically imitate typical activities performed by children and has moderate-to-high criterion-related validity *(rp = 0.78, 95%* confidence interval *[CI] [0.72-0.85])* for estimating $\dot{\text{V}}$O_2max_.^36^ The 20mSRT is traditional,^37^ simple,^38^ and can be used to test large groups of children simultaneously,^39^^,^^40^ it is a good tool for population-based health surveillance and monitoring. Tomkinson et al. provided the most up-to-date and comprehensive and international norms for 20mSRT based on 1,142,026 children and adolescents aged 9–17 years from 50 countries.^41^ This study found that, except for girls aged 12–17 years, the 20mSRT performance of Chinese children and adolescents was worse than the international standards.^41^

Controlling for gender, urban, and rural factors, participants aged 7–18 years, the $\dot{\text{V}}$O_2max_ Z-score of the normal WC group was significantly higher than the high WC group and the very high WC group (*P* < 0.05). Our results were in common with previous studies. The high WC was negatively correlated with cardiopulmonary health.^42^, ^43^, ^44^ In the present study, we also observed a similar association between the WC Z-score and CRF in males and females. $\dot{\text{V}}$O_2max_ Z-score of the normal waist circumference among children and adolescents aged 7–18 years was significantly higher than the high waist circumference.

Similar to the above research, controlling for gender differences, the normal WC group showed better CRF levels than the high WC group. A comparative study of WC and 20mSRT-Z found that 20mSRT-Z in the normal waist circumference group (WC < percentile 75) was higher than that in the high waist circumference group (WC ≥ percentile 75 and < percentile 90) and the very high waist circumference group (WC ≥ percentile 90)of children and adolescents aged 7–18 years.^28^ Previous studies have shown that there was a negative correlation between WC, 20mSRT, and cardio-metabolic risk factors.^45^ Buchan et al. discussed the role of WC and CRF in children and adolescents cardiovascular disease (CVD), identifying a significant negative correlation.^32^ A physical health survey of adolescents in South Africa showed a negative correlation between high WC and cardiopulmonary function, and found that high WC was a risk factor of CVD.^46^ Moreover, some studies found that the accumulation of fat in the waist has a negative effect on lung ventilation function.^47^^,^^48^

The results in this study were basically consistent with those of the above studies, which also verified the negative correlation between high waist circumference and CRF. A study found that variability in daily physical activity (PA) behaviors and sedentary time (min·day^−1^) was associated with WC [sedentary time(+), light physical activity(−)] and CRF [sedentary time(−), moderate-to-vigorous-intensity physical activity (MVPA) (+)].^49^ Thus, it was recommended that children and adolescents should carry out at least 60 min of MVPA in aerobic activities every day to improve CRF according to the physical activity guidelines issued by the World Health Organization (WHO).^50^ But in fact, it was reported that only 30% of Chinese children and adolescents met these recommended guidelines.^51^ Another finding of this study, controlling for gender, urban, and rural factors, children and adolescents aged 10–12, 13–15, and 16–18 years old, the $\dot{\text{V}}$O_2max_ Z-score of the normal WC group was significantly higher than the very low WC group (*P* < 0.05). A study of children aged 8–17 years found that WC could predict CRF. WC was shorter, CRF was better.^52^ The above research was consistent with the view of this study.

### 4.1 Strengths and limitations

Many previous studies have compared waist circumference and cardiorespiratory fitness in children and adolescents, nevertheless, their data was small and unrepresentative. The strengths of this study were the large sample data (N = 92,574) for WC and useful 20mSRT of children and adolescents in six major administrative regions of China.

This study has some limitations that need to be paid attention to. Firstly, the evaluation of $\dot{\text{V}}$O_2max_ in this study is obtained by the indirect test of 20mSRT, which is deviated from the gold standard. Though 20mSRT is traditional,^37^ simple,^38^ and can be used to test large groups of children simultaneously,^39^^,^^40^ it is a field-based estimate of CRF and not a direct measure of CRF. Secondly, only urban and rural factors were controlled, factors such as biological maturity were not considered. However, although biological maturity might impact CRF, particularly in girls, the norms were age- and gender-specific, which may have reduced the influence of growth and development in adolescence to some extent. At last, this was a cross-sectional survey that can not provide longitudinal data to conclude the causality of the correlation between waist circumference and CRF.

## 5 Conclusions

The study showed that CRF presented a trend of a slight rise first and then rapid decline with WC increased in children and adolescents (i.e., an inverted U-shaped curve). The reasons for the research results need to be further investigated and analyzed. In addition, effective measures should be taken to improve CRF and reduce obesity in children and adolescents. More studies are needed to provide a scientific foundation and theoretical basis for controlling waist circumference and improving physical health in children and adolescents.

## Funding

This work was supported by “the Shanghai Philosophy and Social Sciences Planning Office (Award No.:2020BTY001)".

## Authorship contribution statement

**Yuan Liu**: Approval of the version of the manuscript to be published (the names of all authors must be listed), Writing-original draft, Formal analysis, Conceptualization, Writing - review & editing. **Xiaojian Yin**: Approval of the version of the manuscript to be published (the names of all authors must be listed), Writing - review & editing, conceptualization. **Feng Zhang**: Approval of the version of the manuscript to be published (the names of all authors must be listed), Writing - review & editing. **Yuqiang Li**: Approval of the version of the manuscript to be published (the names of all authors must be listed), Writing - original draft. **Cunjian Bi**: Approval of the version of the manuscript to be published (the names of all authors must be listed), Data curation. **Yi Sun**: Approval of the version of the manuscript to be published (the names of all authors must be listed), Formal analysis. **Ming Li**: Approval of the version of the manuscript to be published (the names of all authors must be listed), Formal analysis. **Ting Zhan**g: Approval of the version of the manuscript to be published (the names of all authors must be listed), Data curation.

## Declaration of competing interest

The authors declare no conflicts of interest.
